# Effect of COVID-19 on risk spillover between fintech and traditional financial industries

**DOI:** 10.3389/fpubh.2022.979808

**Published:** 2022-09-07

**Authors:** Haiyang Zhou, Shuping Li

**Affiliations:** ^1^School of Management Science and Engineering, Shandong University of Finance and Economics, Jinan, China; ^2^Jinan Rural Commercial Bank Co., Ltd., Jinan, China

**Keywords:** COVID-19, risk volatility, fintech, traditional finance, MMV-MFDFA

## Abstract

COVID-19 has affected China's financial markets; accordingly, we investigate the effect of COVID-19 on the risk spillover between fintech and traditional financial industries. Using data from April 25, 2012 to April 22, 2022, which we divide into two parts (before and during the COVID-19 periods), we model the dynamic risk spillover relationship following the DCC-GARCH-BEKK and MMV-MFDFA methods. The results show that: (1) The dynamic relationship between fintech and traditional finance is almost positive most of the time, and the dynamic correlations between fintech and realty (real estate development and operation) are the largest. The dynamic linkage between fintech and traditional finance declines after the COVID-19 outbreak. (2) There exists a risk spillover from fintech to every type of bank before and during the COVID-19 periods. Notably, the risk spillover effect of fintech to large state-owned banks and city commercial banks is the largest separately before and during the COVID-19 periods. Meanwhile, there exist a two-way risk spillover between fintech and almost all other traditional financial industries before and during the COVID-19 periods. (3) Owing to the COVID-19 pandemic, the risk spillover relationship, which is in pairs and in the system become more complex. (4) Regarding the whole system, the correlation in the system is anti-persistent most of the time. Moreover, there are large fluctuations and more complex characteristics during the COVID-19 outbreak. However, the whole system was smooth most of the time before the outbreak of the COVID-19 pandemic.

## Introduction

Fintech plays an important role in China's financial innovation, which is of great significance to the development of China's financial industry and the construction of digital China. Traditional financial institutions have gradually become an important subject of fintech innovation. Fintech enhances linkages among financial institutions, and increases the possibility of risk contagion. Fintech makes multiple risks such as traditional financial risk, new technology risk and systemic risk intertwined. Their abruptness, complexity, intersectionality and infectivity are also more prominent. And the initial shocks from fintech will have a greater probability to evolve into systemic risk.

The COVID-19 outbreak poses a serious threat to human life and health, disrupts the trajectory of economic development and threatens the stability of the financial system (1). Financial markets are volatile under the effect of the COVID-19 pandemic. China's Shanghai Composite Index fell by 7.72% on February 3, 2020, which was the largest one-day drop in nearly 5 years after the stock market crash. Fintech has become a major part of China's financial market. Therefore, the effect of COVID-19 may not only increase risks in fintech and traditional financial markets but also change risk spillovers between financial markets and the entire financial system. It is crucial to study the effect of COVID-19 on risk spillovers in fintech and traditional financial markets, which can help prevent and resolve major risks and further improve market resilience in the face of various unexpected events.

Scholars have examined the effect of public emergencies on economic and financial operations. COVID-19 has significantly affected the financial system. Some scholars examine the effect of the pandemic on financial markets, including Chinese stock markets and their various stock sectors indices. Bouri et al. (2) examine the structure change of return connectedness across various assets due to the occurrence of COVID-19. Syed et al. (3) investigate the asymmetric volatility spillover among Chinese stock market sectors during the outbreak of the COVID-19. Abuzayed et al. (4) investigate systemic distress risk spillover between the global stock market and individual stock markets in the countries most affected by the COVID-19. Topcu and Gulal (5) found that the negative effect of COVID-19 on emerging stock markets is gradually diminishing. From a regional perspective, emerging stock markets in Asia are the most negatively affected, whereas those in Europe are the least affected. Goodel (6) analyzes how COVID-19 affect financial markets and institutions, both directly and indirectly. Regarding the relationship between COVID-19 and financial risks, Rizwan et al. (7) evaluate the changes in systemic risk in eight countries, including Canada, during the global financial crisis and COVID-19 periods, and conclude that systemic risk values rose significantly in March, 2020 and peaked in mid-to-late March in all countries during the COVID-19 pandemic, and there are many other scholars explored the effects of COVID-19 on markets (8–18). Some scholars examine the relationship between fintech/cryptocurrencies and financial markets around the COVID-19 outbreak. Muhammad et al. (19) consider bitcoin as a haven for Australia's main stock index during the first and second waves of the COVID-19. Federico (20) studies the impact of bank investment in fintech companies on stock returns. Kumar et al. (21) investigate how cryptocurrencies interact and whether they have clear leaders, paying particular attention to differences in investment horizons and how relationship structures evolve in time. Man et al. (22) study the asymmetric efficiency of cryptocurrency. The COVID-19 epidemic has adversely affected the efficiency of the four cryptocurrencies. Bouri et al. (23) reveal the hedging and safe-haven nature of eight cryptocurrencies against declines in the S&P 500 and its 10 stock sectors. Le et al. (24) investigated whether COVID-19 changes spillover patterns between fintech and other asset classes; the results demonstrate that innovative technology products, as represented by a financial technology index (KFTX) and Bitcoin, were highly susceptible to external shocks.

Many scholars have examined risk spillovers in traditional finance. However, few have examined the risk spillover of fintech. Peer-to-peer lending, electronic payments, crowdfunding, cryptocurrency, and other technological financial innovations are all included in the fintech industry. Fintech and traditional finance compete and collaborate in similar market segments and business areas (25–27). Specifically, fintech hinders the improvement of the banking industry's cost efficiency and promotes the credit supply of micro enterprises in banks; however, it has heterogeneous effects on the profitability of different types of commercial banks (28, 29). Based on the above research, the risk transmission of fintech has also been studied further, mainly including internal and external transmission. Regarding the internal transmission mechanism of fintech risk, the rapid expansion of the fintech industry has driven the emergence of new risks (30). Some scholars have conducted qualitative studies on potential risk types in the fintech industry (31), such as credit, liquidity and operational risk (32), new fraud risk (33), network security, and privacy risk (34). Regarding quantitative analysis, Guo et al. (35) conducte a quantitative study on the credit risk of P2P lending market based on transaction data. Ma et al. (36) analyze the default risk of P2P loans based on mobile phone usage data. Troster et al. (37) predicte the risk of bitcoin cryptocurrency. The risks in the fintech industry are higher than those in traditional and Internet finance (38), and heterogeneity exists among different types of fintech platforms (39). Regarding fintech risk external transmission mechanism, fintech activities may exacerbate risk contagion and asset volatility in the financial system, thereby undermining financial stability. When Internet finance is at extreme risk, there is an obvious risk spillover effect on traditional finance (40). There are multiple connections between fintech companies and traditional financial institutions. Furthermore, the inherent risks of fintech companies may spillover to traditional financial institutions, causing systemic risks. Li et al. (41) assert that the degree of spillover of American fintech companies on the systemic risk of financial institutions is positively correlated with the increase in the systemic risk of financial institutions. Fintech has a heterogeneous effect on the risk-taking behavior of commercial banks (42–45). Le et al. (46) examine the network connectivity and spillover effects between fintech and green bonds and cryptocurrency.

According to the method, the multivariate GARCH model can be used to not only evaluate the fluctuation aggregation characteristics of multiple time series but also effectively evaluate the correlation between different variables. Engle (47) proposed the DCC-GARCH model, which can be used to examine the dynamic time-varying correlation between different time series, accurately captures the correlation between time series, and effectively evaluates the long-term change of correlation. The main advantages of the DCC-GARCH model are the positive definiteness of the conditional covariance matrices and the model's ability to estimate time-varying volatilities, covariances, and correlations among the assets in a parsimonious way (10). DCC-GARCH has been widely used (48–50). We employ DCC-GARCH model to study the dynamic correlation between fintech and traditional financial industry. In order to measure the size and direction of risk spillovers, we further employ GARCH-BEKK model to study the risk spillover between fintech and traditional finance. Many scholars use GARCH-BEKK model to study risk spillover (51–53). The MMV-MFDFA method which is newly proposed by Fan et al. (54) measures the internal fluctuation of the system from the time and fluctuation dimensions, and it is also used in stock market (55, 56).

According to the above discussion, there is no information on the effect of COVID-19 on the risk spillover of fintech and the entire system. This study makes the following contributions: This is the first study to investigate the risk spillover between fintech and traditional finance, the risk spillover in pairs in the system, and the risk spillover of the whole system before and during COVID-19, expanding the existing research boundary; this study aims to investigate the effect of COVID-19 on the risk spillover of fintech and the entire financial system. We further categorize banks to examine the risk spillover of fintech to different types of banks. The findings demonstrate that fintech has different risk spillover effects on different types of banks. In terms of supervision practice, we can monitor and give warning according to the calculation results of risk spillover intensity. This is also the first study on risk spillover in fintech and traditional financial system that follows the new MMV-MFDFA method. The results obtained from this study not only help enrich the research on the mechanism of public emergencies affecting financial stability, but also provide suggestions for regulators to prevent systemic financial risks.

The rest of this article is organized as follows: Section Methodology describes the DCC-GARCH-BEKK and MMV-MFDFA methods. Section Data describes the data, Section Empirical analysis focuses on the empirical analysis results and presents related discussions, and Section Conclusion and implications presents the conclusions.

## Methodology

### DCC-GARCH-BEKK

The DCC-GARCH model is expressed as follows.


(1) $$\begin{array}{r} \gamma_{i,t}=\beta_{0}+\Sigma_{k=1}^{L}\beta K\gamma_{it-k}+\varepsilon_{i} \end{array}$$

(2) $$\begin{array}{r} \varepsilon_{i}I_{t-1}\sim N\left(0,H_{t}\right) \end{array}$$

(3) $$\begin{array}{r} H_{t}=D_{t}R_{t}D_{t} \end{array}$$

(4) $$\begin{array}{r} D_{t}=diag\text{(}\sqrt{h_{1t}},...,\sqrt{h_{kt}}\text{)} \end{array}$$

(5) $$\begin{array}{r} R_{t}=Q_{t}^{*-1}Q_{t}Q_{t}^{*-1} \end{array}$$

(6) $$\begin{array}{r} Q_{t}^{*}=diag\left(\sqrt{q_{11t}},...,\sqrt{q_{kkt}}\right)Q_{t}={\left(q_{ij}\right)}_{kxk} \end{array}$$

The yield vector of variable i at point t is λ_i, t_=(λ_1, t_... λ_k, t_), the information set at t-1 is I_t−1_, the variance-covariance matrix is H_t_, the diagonal matrix composed of standard deviations calculated by the GARCH model is D_t_, and the dynamic correlation coefficient matrix is R_t_. The multivariate dynamic heteroscedasticity is


(7) $$\begin{array}{r} q_{ij,t}=\bar{p_{ij,t}}+\overset{M}{\underset{m=1}{\sum }}\alpha_{m}\left(\varepsilon_{i,t-1}-\bar{pij}\right)\\ +\overset{N}{\underset{n=1}{\sum }}\theta_{n}\left(q_{ij,t-1}-p_{ij}\right) \end{array}$$

The unconditional correlation coefficients of the DCC model are α_m_ and θ_n_, the lag order is m and n, the influence of the product of lag m-order residuals on the dynamic correlation coefficient is α_m_, and the conditional heteroscedasticity coefficient of the lag n-phase is θ_n_. A positive characterization of H_t_ can be guaranted if the conditions of α_*m*_≥0, θ_*n*_≥0 and $\sum_{m=1}^{M}\alpha_{m}+\sum_{n=1}^{N}\theta_{n}<1$ are satisfied.

Engle and Kroner (57) proposed the GARCH-BEKK model after a positive qualitative adjustment of the matrix, which is mainly used to examine the dynamic distribution of the covariance matrix of various financial markets.


(8) $$\begin{array}{r} RX_{t}=u_{k}+\overset{n}{\underset{i=1}{\sum }}\alpha_{ki}RA_{t-i}+\overset{n}{\underset{i=1}{\sum }}\beta_{ki}RL_{t-i}+\varepsilon_{kt},\\ X=A,L;k=1,2 \end{array}$$

Vector RX_t_= (RA_t_, RL_t_) represents the level of the t period of the two return sequences, u_1_ and u_2_ are the intercept terms of each model, α_1i_, α_2i_... ε_1t_ and ε_2t_ are the corresponding coefficients and residuals of the two mean equations, respectively, and n is the lag order.

For BEKK-GARCH model, the change process of H_t_ is:


(9) $$\begin{array}{r} Ht=C^{T}C+A^{T}\varepsilon t-1\varepsilon_{t-1}^{T}A+B^{T}Ht-1B \end{array}$$

The parameter matrix represents the lower triangular constant matrix and the coefficient matrix of the ARCH and GARCH terms, corresponding to the short-term and long-term fluctuation components, respectively.

### MMV-MFDFA

(x_1k_, x_2k_, …, x_jk_, …, x_pk_) are multivariate time series, k = 1, …, N, and j = 1, …, p, where p denotes the number of variables, and N denotes the length of each variate.

Determine the profile of the jth variate


(10) $$\begin{array}{r} X_{i}^{j}=\overset{i}{\underset{k=1}{\sum }}\left(x_{k}^{j}-\langle x^{\left(j\right)}\rangle \right) \end{array}$$

where 〈*x*^(*j*)^〉 is the average of the time series $X_{i}^{j}$ for *i* = 1, 2, …, N and *j* = 1, 2, …, p.

Divide the time series $X_{i}^{j}$ into Ns = int[N/s] non-overlapping subintervals; all *s* have the same length. The same procedure is followed for the inverse of the time series to obtain the 2N_S_ subintervals.

For each subinterval *v*, we employed the least-squares method to fit the polynomial function. For *v* = *1, 2*, …*, 2Ns*, the local detrended covariance function is


(11) $$\begin{array}{r} f^{2}\left(v,s\right)=\frac{1}{s}\overset{s}{\underset{t-1}{\sum }}||\left(X_{lv+t}^{1},X_{lw+t}^{2},\ldots ,X_{lv+t}^{P}\right)-\left({\tilde{X}}_{1}^{v},{\tilde{X}}_{2}^{v},\ldots {\tilde{X}}_{p}^{v}\right)\\ ||\cdot ||\left(Y_{lv+t}^{1},Y_{lw+t}^{2},\ldots ,Y_{lv+t}^{P}\right)-\left({Ỹ}_{.,1}^{v},{Ỹ}_{.,2}^{v},\ldots {Ỹ}_{.,p}^{v}\right) \end{array}$$

${\tilde{X}}_{.,i}^{V},{\tilde{Y}}_{.,i}^{V}$ (*i*=1,2......p) represents the fitting polynomial corresponding to the *i*th variable in subinterval *v*, ||∙|| represents the Euclidean norm, and


(12) $$\begin{array}{r} \left||X-Y||=||\left(x_{1},x_{2},\ldots ,x_{n}\right)-\left(y_{1},y_{2},\ldots ,y_{n}\right)|\right|\\ =\sqrt{\overset{n}{\underset{i=1}{\sum }}{\left(x_{i}-y_{i}\right)}^{2}} \end{array}$$

The *q*-order fluctuation function *F(q,s)* of the multivariate time series is calculated as follows, when *q* is a real number and does not equal zero,


(13) $$\begin{array}{r} F_{xy}^{q}\left(s\right)={\left\{\frac{1}{2N_{s}}\overset{2N_{s}}{\underset{v=1}{\sum }}\left[F^{2}{\left(s,v\right)}^{q/2}\right]\right\}}^{1/q} \end{array}$$

When *q*=*0*, the fluctuation function is reported as follows.


(14) $$\begin{array}{r} F_{{}_{xy}}^{0}\left(s\right)=\exp\left\{\frac{1}{4N_{s}}\overset{2N_{s}}{\underset{v=1}{\sum }}\left[\ln\;F^{2}\left(s,v\right)\right]\right\} \end{array}$$

Calculate the fluctuation function *F*_*q*_*(s)* corresponding to the different time scales *s*. The fluctuation function *F*_*q*_*(s)* and time scale *s* have the following power law relationship:


(15) $$\begin{array}{r} F_{xy}^{q}\left(s\right)\;˜\;s^{H_{xy}\left(q\right)} \end{array}$$

We calculated the *q*-order fluctuation function *F*_*q*_*(s)* of the points in each window and a quasi-continuous variation in *H*_*xy*_*(q)* with scale *s*, which is represented by the binary function *H*_*xy*_*(q)*. The graph of this binary function is the Hurst surface, and the height of each point represents the value of the generalized cross-correlation index *H*_*xy*_*(q)* corresponding to *(q,s)*. As the center (average scale) and window range of the sliding fitting window change constantly, *s*=*(a*+*b)/2* is used to represent the fitting window *s* to better display the Hurst surface. Therefore, the generalized dependent Hurst surfaces are defined as:


(16) $$\begin{array}{r} h\left(q,s\right)=\frac{\log\left[F\left(q,s\right)\right]}{\log\left(s\right)} \end{array}$$

where *F(q,s)* is the *q*-order fluctuation function for the points falling into the window. As the fluctuation functions *F(q,s)* are presented in log-log coordinates, the moving fitting window expands logarithmically.

## Data

We use the daily closing prices of the fintech and traditional financial indices, covered from Apr 25, 2012 to Apr 22, 2022. We divide the sample into two parts: the data from Jan 20, 2020 to Apr 22, 2022, as the sample during COVID-19, and the rest as the sample before COVID-19. Data is obtained from the Wind database. We calculate the returns of the fintech and traditional financial industries using the logarithmic difference in the daily closing prices:


$$r_{\text{t}}=ln\left(P_{\text{t}}\right)-ln\left(P_{\text{t}-1}\right)$$

Table 1 shows the descriptive statistics of all the indices. The mean values of the seven samples are close to 0 and the standard deviations are larger than 0. The minimum values of all the returns are close to−4, and the maximum value of fintech is close to 3, while that of traditional finance is close to 4. The skewness values of the returns are not 0, and the kurtosis values are all larger than 3. The Jarque-Bera test rejects the null hypothesis of a normal distribution.

**Table 1:** Descriptive statistics.

	**Fintech**	**Bank**	**National joint stock bank**	**Large state-owned bank**	**City commercial bank**	**Insurance**	**Trust**	**Realty**
Mean	0.0172	0.0157	0.0175	0.0108	0.0195	0.0138	0.0064	0.0075
Median	0.0189	−0.0186	−0.0207	−0.0052	−0.0133	−0.0145	−0.0050	0.0049
Maximum	3.2602	3.7508	3.9828	4.0119	4.1436	4.0788	4.1522	4.0839
Minimum	−4.2550	−4.5618	−4.5655	−4.5542	−4.5629	−4.4337	−4.5835	−4.3322
Std. dev.	0.9313	0.6509	0.7198	0.5727	0.7290	0.8396	1.0965	0.8078
Skewness	−0.4413	0.1223	0.1944	0.0098	0.2772	0.1740	−0.0923	−0.5935
Kurtosis	5.0607	9.8544	8.4009	14.6441	10.3031	6.4854	6.6100	7.5958
Jarque–Bera	509.0658	4765.0337	2969.9827	13733.6133	5433.5370	1242.7496	1323.5217	2282.1186

## Empirical analysis

### Impulse response

**Figure 1:**
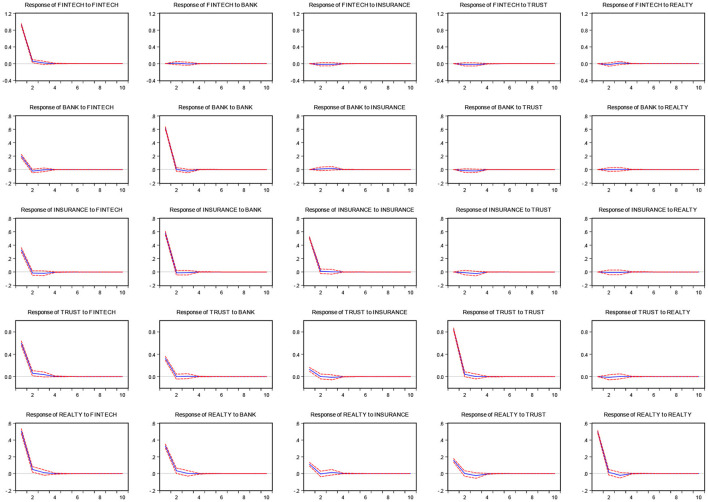
Impulse response analysis.

Figure 1 shows the impulse response results for the entire sample period. The figure shows that the responses of fintech to traditional financial industries are small. While the responses of traditional financial industries to fintech are large, the response of realty to fintech is the largest. There are deviations of nearly 2, 4, 0.6, and 6 units in the first phase for the responses of banks, insurance, trust, and realty, respectively. The response of banks to insurance is also large, with a deviation of nearly 6 units in the first phase. The response of trust to banks and insurance is small, with a deviation of nearly 0.4 and 0.2 units, respectively, in the first phase. There are also some responses of realty to bank, insurance, and trust—a deviation of nearly 4, 2, and 2 units, respectively, in the first phase.

### The dynamic correlation analysis

**Figure 2:**
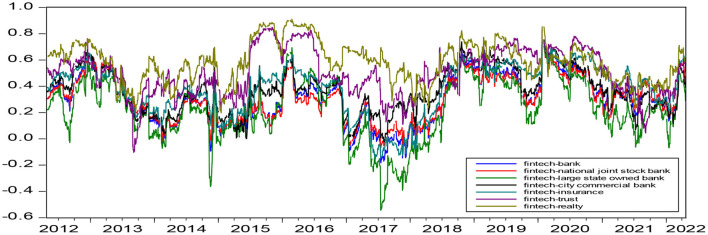
Dynamic correlation coefficient diagram.

Figure 2 shows that: (1) The dynamic correlation coefficient trend between fintech and each traditional financial industry is generally significantly similar, especially for the trend of fintech and national joint stock banks, fintech and large state-owned banks, and fintech and city commercial banks, which are highly consistent with those of fintech and banks. During the dynamic correlations between fintech and the three kinds of banks, that of city commercial banks is the largest and that of large state-owned banks is the smallest before the COVID-19 period. City commercial banks and national joint stock banks are basically consistent and still larger than those of large state-owned banks during the COVID-19 period. (2) The dynamic relationship between fintech and traditional finance is almost positive for most of the time. (3) Among all dynamic correlations, that between fintech and realty is the largest. (4) The dynamic linkage between fintech and traditional finance declined after the COVID-19 outbreak.

### Risk spillover analysis

We divide the entire sample into two parts, pre-pandemic and during-pandemic, and examine the risk spillover between fintech and traditional finance during the two periods separately using the GARCH-BEKK method to further examine the effect of COVID-19 on risk spillover. Tables 2–5 show the results.

**Table 2:** Conditional variance estimation between fintech and banks.

	**Fintech-bank**		**Fintech-national joint stock bank**		**Fintech-large state-owned bank**		**Fintech-city commercial bank**	
	**Pre-pandemic**	**During pandemic**	**Pre-pandemic**	**During pandemic**	**Pre-pandemic**	**During pandemic**	**Pre-pandemic**	**During pandemic**
c_11_	0.0725***	0.0726***	0.0774***	0.0766***	0.1030***	0.1134***	0.0634***	−0.0707***
c_21_	−0.0176	−0.0053	−0.0119	−0.0122	−0.0450***	−0.0539***	0.0078	−0.0176
c_22_	0.0801***	0.1031***	0.0732***	0.0920***	0.0688***	0.0770***	0.0927***	0.1020***
a_11_	0.2173***	0.1919***	0.2210***	0.1967***	0.2065***	0.1917***	0.2092***	0.1768***
a_12_	−0.0487***	−0.0537**	−0.0500**	−0.0582***	−0.0628***	−0.0546***	−0.0373**	−0.0659***
a_21_	−0.0831	0.0328	−0.0554	0.0300	−0.1291***	−0.1144**	−0.0742*	0.0455
a_22_	0.2998***	0.3417***	0.2875***	0.3255***	0.3887***	0.4183***	0.2935***	0.3433***
b_11_	0.9717***	0.9791***	0.9724***	0.9776***	0.9672***	0.9658***	0.9735***	0.9828***
b_12_	0.0100***	0.0145***	0.0104***	0.0136**	0.0147***	0.0156***	0.0085**	0.0171***
b_21_	0.0330**	−0.0068	0.0193	−0.0047	0.0730***	0.0814***	0.0261*	−0.0145***
b_22_	0.9450***	0.9241***	0.9531***	0.9385***	0.9068***	0.8872***	0.9462***	0.9268***

***, **, and * denote the significance of the expression at the 1%, 5%, and 10% levels, respectively.

**Table 3:** Risk spillover between fintech and banks.

**Fintech-bank**			**Fintech-national**			**Fintech-large**			**Fintech-city**		
			**joint stock bank**			**state-owned bank**			**commercial bank**		
	**Pre-pandemic**	**During pandemic**		**Pre-pandemic**	**During pandemic**		**Pre-pandemic**	**During pandemic**		**Pre-pandemic**	**During pandemic**
From fintech to bank	F(2,*) = 4.3304**	F(2,*) = 4.1481**	From fintech to national joint stock bank	F(2,*) = 3.8894**	F(2,*) = 3.7772**	From fintech to large state-owned bank	F(2,*) = 9.4587***	F(2,*) = 6.5234***	From fintech to city commercial bank	F(2,*) = 3.0648**	F(2,*) = 17.1160***
From bank to fintech	F(2,*) = 2.1154	F(2,*) = 0.2898	From national joint stock bank to fintech	F(2,*) = 0.8603	F(2,*) = 0.5454	From large state-owned bank to fintech	F(2,*) = 11.9764***	F(2,*) = 6.1421***	From city commercial bank to fintech	F(2,*) = 1.6569	F(2,*) = 0.3114

***, **, and * denote the significance of the expression at the 1%, 5%, and 10% levels, respectively.

**Table 4:** Conditional variance estimation between fintech and other traditional financial industries.

	**Fintech-insurance**		**Fintech-trust**		**Fintech-realty**	
	**Pre-pandemic**	**Pandemic**	**Pre-pandemic**	**Pandemic**	**Pre-pandemic**	**Pandemic**
c_11_	0.0821***	0.0840***	0.0804***	0.0688***	0.0396	0.0276
c_21_	−0.0127	−0.0026	0.0157	0.0120	0.0073	−0.0444
c_22_	0.0578***	0.0688***	−0.0949^***^	0.0932***	0.0646***	0.0408
a_11_	0.2229***	0.2105***	0.1932***	0.1639***	0.1147***	0.1037***
a_12_	−0.0374	−0.0516*	−0.0690**	−0.1084***	−0.1321***	−0.1534***
a_21_	−0.0231	0.0321	0.0205	0.0698**	0.1168***	0.1317***
a_22_	0.2315***	0.2582***	0.3189***	0.3762***	0.3439***	0.3726***
b_11_	0.9700***	0.9721***	0.9780***	0.9847***	0.9968***	0.9977***
b_12_	0.0057	0.0096	0.0160**	0.0247***	0.0282***	0.0323***
b_21_	0.0129	−0.0017	−0.0018	−0.0168*	−0.0326***	−0.0346***
b_22_	0.9714***	0.9638***	0.9466***	0.9282***	0.9368***	0.9283***

***, **, and * denote the significance of the expression at the 1%, 5%, and 10% levels, respectively.

**Table 5:** Risk spillover between fintech and other traditional financial industries.

**Fintech-insurance**			**Fintech-trust**			**Fintech-realty**		
	**Pre-pandemic**	**pandemic**		**Pre-pandemic**	**pandemic**		**Pre-pandemic**	**Pandemic**
From fintech to insurance	F(2,*) = 1.2941	F(2,*) = 1.4484	From fintech to trust	F(2,*) = 3.1054**	F(2,*) = 8.9049***	From fintech to realty	F(2,*) = 17.7402***	F(2,*) = 21.6478***
From insurance to fintech	F(2,*) = 1.5345	F(2,*) = 1.5365	From trust to fintech	F(2,*) = 1.2894	F(2,*) = 3.4190**	From realty to fintech	F(2,*) = 10.1241***	F(2,*) = 15.8939***

***, **, and * denote the significance of the expression at the 1%, 5%, and 10% levels, respectively.

Tables 2, 3 show the results of the conditional variance estimation and risk spillover, respectively. The results show that there exists a risk spillover from fintech to the banking industry before and during the COVID-19 outbreak. Moreover, the risk spillover effect does not change significantly. Further, we classify the bank industry into three categories, national joint stock banks, large state-owned banks, and city commercial banks, and investigate the risk spillover between fintech and the three types of bank industries. Regarding the risk spillover between fintech and national joint stock banks, there exists only a risk spillover from fintech to the national joint stock bank before and during the COVID-19 pandemic. However, it does not change significantly. There further exists a two-way risk spillover between fintech and large state-owned banks; the risk spillover effect decreases before the COVID-19 outbreak. There also exists risk spillover from fintech to city commercial bank before and during the COVID-19 pandemic; the risk spillover increases during the period before COVID-19.

Further, we compare the risk spillover effect of fintech on the three types of banks before and during the pandemic. Fintech has the largest risk spillover to large state-owned banks. The effect on state-owned joint stock banks and city commercial banks have insignificant difference before the pandemic. Among the three types of banks, fintech has the largest risk spillover to city commercial banks during the COVID-19 pandemic. Only state-owned banks have a risk spillover effect on fintech; therefore, we did not compare the risk spillover sizes of various banks on fintech before and during the pandemic.

This is mainly because fintech has formed a deep integration with various banks in business cooperation, technology outsourcing, and data sharing. Fintech risk spillover on to various banks to different degrees through these channels. First, the risk spillover from fintech to large state-owned banks is greater owing to their larger size and stronger business ties with the fintech industry. After the COVID-19 outbreak, large state-owned banks have more transparent information disclosure, a higher degree of supervision, a better risk assessment mechanism, a more rational application of fintech, and reduce the risk spillover of fintech to large state-owned banks. Second, COVID-19 has severely damaged market confidence, aggravated social panic, aggravated asset price volatility, worsened investment returns, and increased risk-taking by banks. Meanwhile, COVID-19 has negatively affected household consumption, and a large number of banks have launched consumer loans. However, large state-owned banks have more sufficient economic resources for the application and deployment of fintech and have a stronger ability to deeply integrate information technology and better identify risky customers. Some high-risk projects and customers turn to small and medium-sized banks, such as city commercial banks; therefore, the risk spillover from fintech to city commercial banks is the largest after the pandemic. Third, large state-owned banks have extensive physical outlets and numerous customers, and most of their customers are state-owned enterprises and large customers with low flexibility in deposit interest rates (58). However, the customer groups of small and medium-sized banks and the fintech industry relatively overlap. Moreover, most of them are small and micro enterprises and long-tail groups with high interest rate sensitivity. Finally, the large business lines of big state-owned banks, integrated with the diversified ways in which big banks supplement their capital, give them relatively strong bargaining power over the cost of capital. Therefore, after the COVID-19 outbreak, compared with large state-owned banks, the risk spillover of fintech to small and medium-sized banks, such as city commercial banks, is greater.

Tables 4, 5 clearly show that there is no risk spillover between fintech and insurance, both before and during COVID-19 pandemic. The tables show that there exists risk spillover from fintech to trust before the COVID-19 pandemic and that there exists two-way risk spillover between fintech and trust during the COVID-19 pandemic. They further show that the effect of fintech on trust increase during the COVID-19 pandemic than before the outbreak of the pandemic. There also exists two-way risk spillover between fintech and realty before and during the COVID-19 pandemic. Moreover, the effect between them increases during the COVID-19 pandemic than before the outbreak of the pandemic.

### Risk spillover in pairs in the system

Table 6 shows the results of the risk spillover in pairs in the system. We deleted the conditional variance estimation table due to lack of space. The table clearly shows that there exists two-way risk spillover between fintech and insurance, fintech and realty, and banks and insurance before the COVID-19 outbreak. There also exists a one-way risk spillover from trust to fintech and from bank to realty before the COVID-19 outbreak. However, the risk spillover relationship in pairs in the system has changed. Only the risk spillover relationships between insurance and banks, insurance and realty are two-way. There exists one-way risk spillover from fintech to banks, from fintech to insurance, from trust to realty, from trust to insurance, from realty to banks and from trust to banks. Owing to the COVID-19 pandemic, the risk spillover relationship has become more complex.

**Table 6:** Risk spillover in pairs in the system.

	**Pre-pandemic**	**Pandemic**
From fintech to bank	F(2,*) = 0.6543	F(2,*) = 5.2126***
From bank to fintech	F(2,*) = 0.7594	F(2,*) = 0.0008
From fintech to insurance	F(2,*) = 4.5477**	F(2,*) = 12.3252***
From insurance to fintech	F(2,*) = 3.7077**	F(2,*) = 0.1866
From fintech to trust	F(2,*) = 1.3406	F(2,*) = 0.7250
From trust to fintech	F(2,*) = 2.4656*	F(2,*) = 0.1199
From fintech to realty	F(2,*) = 10.2867***	F(2,*) = 1.0417
From realty to fintech	F(2,*) = 10.3727***	F(2,*) = 0.9577
From bank to insurance	F(2,*) = 2.3499*	F(2,*) = 33.4541***
From insurance to bank	F(2,*) = 3.3124**	F(2,*) = 88.3296***
From bank to trust	F(2,*) = 0.3371	F(2,*) = 0.7077
From trust to bank	F(2,*) = 0.0200	F(2,*) = 3.1436**
From bank to realty	F(2,*) = 2.9235*	F(2,*) = 2.0239
From realty to bank	F(2,*) = 1.5863	F(2,*) = 5.0517***
From insurance to trust	F(2,*) = 0.1322	F(2,*) = 1.8449
From trust to insurance	F(2,*) = 0.1864	F(2,*) = 9.5914***
From insurance to realty	F(2,*) = 1.0526	F(2,*) = 4.4644**
From realty to insurance	F(2,*) = 0.0675	F(2,*) = 8.0871***
From trust to realty	F(2,*) = 1.6842	F(2,*) = 3.2562**
From realty to trust	F(2,*) = 0.8222	F(2,*) = 0.4731

***, **, and * denote the significance of the expression at the 1%, 5%, and 10% levels, respectively.

### Risk spillover of the system

**Figure 3:**
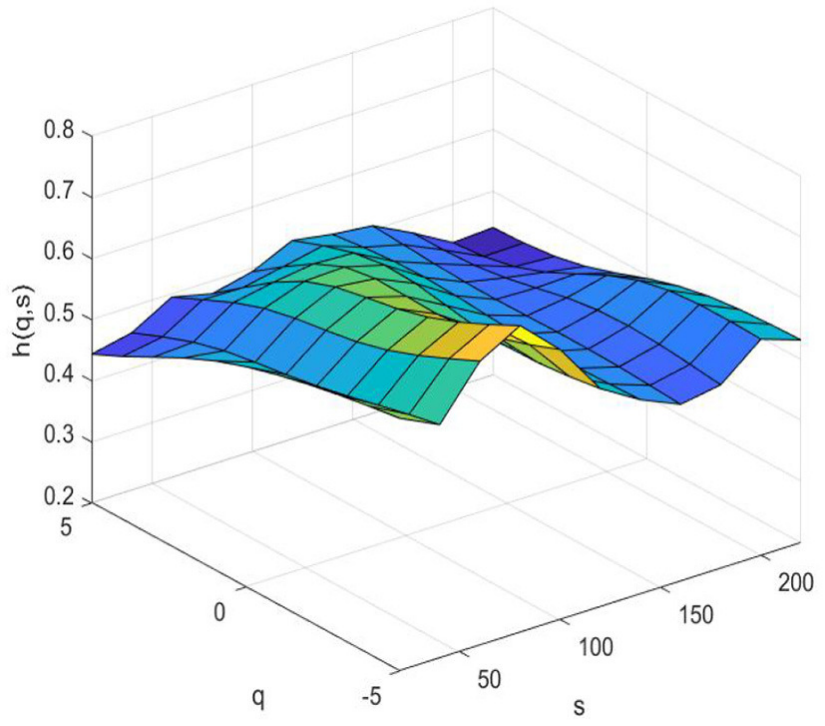
Risk spillover of the system (pre–pandemic).

**Figure 4:**
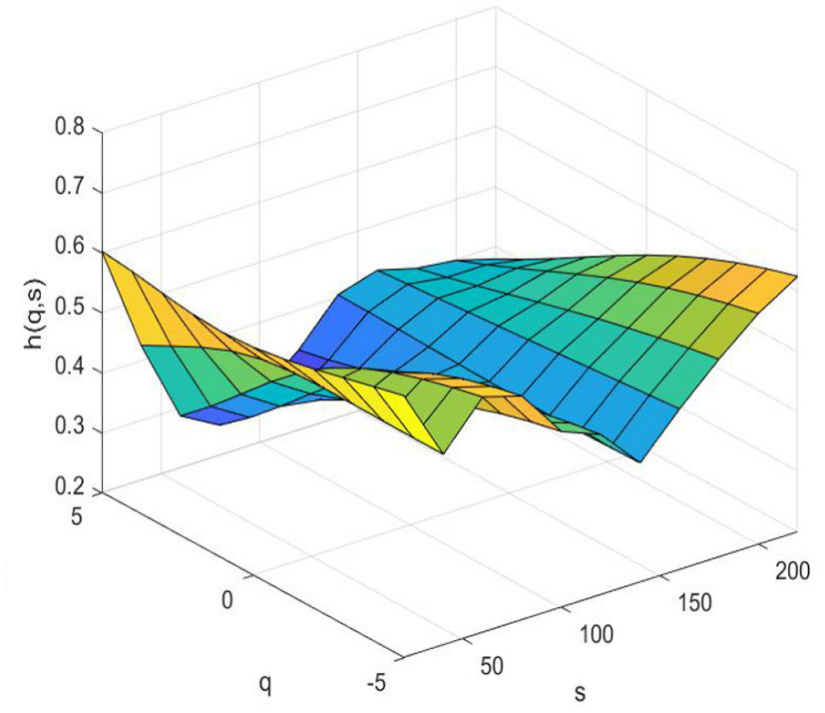
Risk spillover of the system (pandemic).

Figure 3 (pre-pandemic) and 4 (pandemic) show the systemic risk associated with fintech in terms of multifractals by MMV-MFDFA. The Hurst surface fluctuates with changes in the time scale s and fluctuation q dimensions. Figure 3 shows that the Hurst surface is smooth most of the time, and the Hurst exponents fluctuate around 0.5, which demonstrates that the system associated with fintech fluctuates between persistent and anti-persistent with a few small fluctuations before the COVID-19 period. Whereas Figure 4 shows that the Hurst surface has large fluctuations. During the COVID-19 pandemic, most of the Hurst exponents are smaller than 0.5, both in the short and long term, indicating that the correlation in the system is anti-persistent most of the time. It fluctuates considerably, regardless of large or small fluctuations in the system. Volatility is greater in the short term than that in the long term. The reason is that the short-term behavior of the fintech system are susceptible to the influence of external factors such as the COVID-19. The findings also demonstrates that the fintech system is relatively stable when the market environment is relatively stable, However, once there are external emergencies with relatively large influences, the fintech system will fluctuate greatly or even change fundamentally. In other words, the correlation between fintech and traditional financial industries is quite sensitive to external shocks.

## Conclusion and implications

This study adopts DCC-GARCH-BEKK and MMV-MFDFA to explore the risk spillover between the fintech and traditional financial industries in pairs and in the system. The chosen data sample covers the period from April 25, 2012 to April 22, 2022. Our conclusions are summarized as follows.

The dynamic relationship between fintech and traditional finance is almost positive for most of the time and the dynamic correlations between fintech and realty are the largest. We further investigate the risk spillover between fintech and traditional financial industries, risk spillover in pairs in the system, and risk spillover of the entire system before and during the COVID-19 pandemic. The results demonstrate that there exists a risk spillover from fintech to every type of bank before and during the COVID-19 period, while the risk spillover effect of fintech to large state-owned banks and city commercial banks is the largest before and during the COVID-19 period separately. However, only large state-owned banks have a risk spillover to fintech before and during the COVID-19 outbreak. Meanwhile, there exists two-way risk spillover between fintech and other traditional financial industries before and during the COVID-19 pandemic. Owing to the COVID-19 pandemic, the risk spillover relationship in pairs in the system has become more complex. Regarding the entire system, the correlation in the system is anti-persistent most of the time. Moreover, there are large fluctuations and more complex characteristics during the COVID-19 outbreak, whereas the entire system is smooth most of the time before the COVID-19 outbreak.

This findings have important implications for policymakers and researchers. First, owing to the dynamic linkage between fintech and traditional finance, appropriate policies should be implemented in time for individual financial markets to reduce risk contagion caused by the existence of related mechanisms when shocks such as the COVID-19 pandemic occur. Meanwhile, research should consider vulnerable markets more, to reduce the uncertainty of the entire financial system owing to the increased risk in one market. Second, the risk spillover of fintech to city commercial banks is the largest after the pandemic. Accordingly, regulators should reduce the pressure on fintech deployment of small and medium-sized banks, such as city commercial banks, and improve intelligent risk control ability. Third, fintech has a risk spillover effect on most traditional financial industries. Accordingly, regulatory authorities should consider fintech more, improve the construction of supporting regulations, reinforce relevant tracking research and risk assessment, determine risks, seek countermeasures in advance as far as possible, and reduce the time lag of supervision. Finally, the regulatory authorities should make full use of the advantages of big data, reinforce the risk information disclosure and sharing mechanism, and ensure that the development of fintech is limited within the basic framework of serving the real economy, to avoid systemic financial risks and prevent the occurrence of systemic financial risks. Last but not the least, for investors, innovative asset represented by fintech index has been proved to increase by the volatility spillovers from other assets during the covid-19 pandemic, so they should not be regarded as safe havens. The results provide important significance for designing effective diversification strategies; These findings provide preliminary evidence that fintech companies are more sensitive to the impact of the COVID-19 pandemic.

Simultaneously, there are some limitations in the research. For example, we did not analyze the dynamic linkage and risk spillover relationship between fintech and rural commercial banks on account of data's unavailability. While rural commercial banks are also an important part of China's banking system. In subsequent studies, we could further extend bank sample size and make more comprehensive and accurate research.

## Data availability statement

The original contributions presented in the study are included in the article/supplementary material, further inquiries can be directed to the corresponding author/s.

## Author contributions

HZ: conceptualization, methodology, data curation, software, formal analysis, and writing-original draft preparation. SL: supervision, validation, investigation, methodology, and writing-review and editing. All authors contributed to the article and approved the submitted version.

## Funding

This research was funded by Shandong Province Key Research and Development Program (Soft Science Project) (No. 2021RKY03052).

## Conflict of interest

Author HZ was employed by Jinan Rural Commercial Bank Co., Ltd. The remaining author declares that the research was conducted in the absence of any commercial or financial relationships that could be construed as a potential conflict of interest.

## Publisher's note

All claims expressed in this article are solely those of the authors and do not necessarily represent those of their affiliated organizations, or those of the publisher, the editors and the reviewers. Any product that may be evaluated in this article, or claim that may be made by its manufacturer, is not guaranteed or endorsed by the publisher.
